# The Serum Profile of Transferrin Isoforms in Pancreatitis

**DOI:** 10.3390/jcm11061638

**Published:** 2022-03-16

**Authors:** Agnieszka Mucha, Malgorzata Zaczek, Michal Kralisz, Ewa Gruszewska, Bogdan Cylwik, Anatol Panasiuk, Lech Chrostek

**Affiliations:** 1 Department of Laboratory Diagnostics, University Clinical Hospital in Bialystok, 15-535 Bialystok, Poland; agnieszka.grytczuk@onet.eu; 2 Department of Gastroenterology, Hepatology and Internal Diseases with the Center of Diagnostics and Endoscopic Treatment, Provincial Welded Hospital in Bialystok, 15-278 Bialystok, Poland; malgorzata_zaczek1@wp.pl (M.Z.); michal.kralisz@gmail.com (M.K.); anatol@panasiuk.pl (A.P.); 3 Department of Biochemical Diagnostics, Medical University of Bialystok, 15-269 Bialystok, Poland; ewa.gruszewska@umb.edu.pl; 4 Department of Pediatric Laboratory Diagnostics, Medical University of Bialystok, 15-274 Bialystok, Poland; cylwikb@umb.edu.pl; 5 Department of Clinical Medicine, Medical University of Bialystok, 15-254 Bialystok, Poland

**Keywords:** pancreatitis, transferrin isoforms, capillary electrophoresis

## Abstract

Total transferrin concentration changes in acute-phase reactions. Additionally, the alteration of transferrin glycosylation in inflammations can occur. The aim of this study is to evaluate the effect of pancreatitis on the serum profile of transferrin isoforms. The tested groups consisted of 84 patients with acute pancreatitis and 42 patients with chronic hepatitis. Transferrin isoforms were analyzed by capillary electrophoresis on a MINICAP electrophoretic system (Sebia, France). There was a significant decrease in the concentration of pentasialotransferrin in both acute and chronic pancreatitis, and a significant increase in tetrasialotransferrin in the acute pancreatitis group when compared to the control group. There were no significant changes in transferrin isoforms between the acute and chronic pancreatitis groups, and between the edematous and necrotizing forms of the disease. Considering the etiology of acute pancreatitis, we noticed higher values of bile acids and γ-glutamyltransferase in acute pancreatitis of alcoholic etiology than that in pancreatitis of other etiologies. In conclusion, the alterations in transferrin isoform profile in acute and chronic pancreatitis are not organ specific. Because similar changes were observed in hepatitis, we can conclude that the serum profile of transferrin isoforms is involved in the pathogenesis of the disease.

## 1. Introduction

It is well known that alterations in protein glycosylation occur in various diseases, including inflammations, which, as a result, cause a quantitative shift in glycoforms of different glycoproteins [1,2]. One such highly variable glycoprotein is transferrin (Tf), whose different isoforms result from variations in the glycosylation of oligosaccharide chains [3]. Additionally, transferrin is a negative acute-phase protein, whose concentration decreases during inflammatory responses [4]. Previously, we reported the changed profile of carbohydrate-deficient transferrin (a sum of the asialo, monosialo and disialo isoforms of transferrin, CDT for short) in pancreatic diseases, including pancreatitis of different etiologies (alcoholic and biliary) [5] and in pancreatic cancers [6]. It has been also shown that transferrin sialylation can be a potential prognostic marker for the severity of acute pancreatitis [7]. In this study, we determine the profile of transferrin isoforms by means of the capillary electrophoresis method, which enables the determination of five different isoforms (asialo-, disialo-, trisialo-, tetrasialo- and pentasialo-transferrin) [8]. We expect to obtain the characteristic profile of Tf isoforms, which is different from that in pancreatic cancers [6], liver [9,10] and rheumatic diseases [11,12].

## 2. Patients and Methods

### 2.1. Patients

The study was carried out on the sera of 84 patients (59 men and 25 women) with acute pancreatitis (mean age: 38 years; range: 23–70 years) and 42 patients (34 men and 8 women) with chronic pancreatitis (mean age: 46 years; range: 19–71 years) admitted to the Department of Gastroenterology, Hepatology and Internal Diseases with the Center of Diagnostics and Endoscopic Treatment (Provincial Welded Hospital in Bialystok, Poland).

### 2.2. Blood Sampling

Blood samples (7 mL) were taken from all patients by vein puncture once after admittance. The blood was allowed to clot. The sera were separated by centrifugation at 1500 g for 10 min at room temperature. All samples were stored at 86 °C until analysis. In addition to sera, portions of each blood sample were subsequently collected into two tubes: one containing anticoagulant (ethylene-diamine tetraacetic acid (EDTA-2K)) for hematological assays (MCV and platelets), and the second containing sodium citrate (0.1 M/L) for coagulation tests [PT, INR].

### 2.3. Diagnosis

The diagnosis of acute pancreatitis (AP) was made on the basis of clinical signs and symptoms (upper abdominal pain, abdominal pain that radiates to the back, tenderness when touching the abdomen, fever, rapid pulse, nausea and vomiting). The diagnosis of chronic pancreatitis (CP) was made on the basis of the following clinical signs and symptoms: upper abdominal pain, abdominal pain that feels worse after eating, losing weight without trying, and oily, smelly stools. To confirm the diagnosis, each patient underwent blood tests (pancreatic enzymes, complete blood count and liver enzymes), abdominal ultrasound, computerized tomography (CT), magnetic resonance imaging (MRI), endoscopic ultrasound, and stool tests). To resolve the diagnostic problems in the bile tract and pancreatic duct, and to remove obstructions, such as gallstones, Endoscopic Retrograde Cholangiopancreatography (ERCP) was recommended. Acute intestinal edematous pancreatitis was observed in 42 AP patients and acute necrotizing pancreatitis in 42 AP patients. The alcoholic etiology of pancreatitis was approved in 21 patients. Other causes of acute pancreatitis were: gallstones, hypertriglyceridemia, drugs and unknown causes. The control subjects (*n* = 30; 16 men and 14 women aged 32 years; range 22–54 years) were recruited from healthy volunteers. The laboratory characteristics of patients and controls are presented in Table 1. Written informed consent was obtained from each patient after the explanation of the nature of the study. The study was approved by the local research ethics committee for Medical University of Bialystok (APK.002.67.2020).

### 2.4. Laboratory Testing

Amylase, lipase, AST, ALT, GGT, ALP, glucose, total bilirubin, total transferrin, C-reactive protein (CRP), cholesterol (Chol), and triglycerides (TG) were determined on a Cobas c501 Analyzer (Hitachi, Tokyo, Japan). The platelet count (PLT) was measured on a Sysmex XS-800i (Sysmex Corporation, Singapore). Prothrombin time (PT) was measured on an STA Compact Max analyzer (Stago) by the viscometric method. Total bile acids (BA) were determined by the enzyme cycling method using the Diazyme Total Bile Acids Assay kit (Diazyme Laboratories, Gregg Court, Poway, CA, USA) on Indiko Plus analyzer (Thermo Fisher Scientific, Vantaa, Finland).

### 2.5. Transferrin Isoforms Testing

The electrophoretic analysis of transferrin was performed on a MINICAP system (Sebia, France), according to the manufacturer’s instructions. The MINICAP CDT reagent kit (Sebia, France) was applied. The MINICAP system uses the principle of capillary electrophoresis (CE) in a free solution. The system performs all sequences automatically to obtain a complete transferrin isoform profile for the quantitative analysis of each fraction. The human serum transferrin isoforms were separated in an alkaline buffer (pH 8.8) into five major fractions according to their sialylation level: asialotransferrin, disialotransferrin, trisialotransferrin, tetrasialotransferrin and pentasialotransferrin.

### 2.6. Statistical Analysis

Results are expressed as means and standard deviations. The differences between tested and control groups were evaluated by Mann–Whitney U-test. We considered *p* values < 0.05 as statistically significant.

## 3. Results

The results of tests in the controls and acute and chronic pancreatitis are presented in Table 1. The mean values of MCV, PT, CDT, AST, ALT, amylase, lipase, ALP and CRP were significantly higher, but the mean values of 5-sialoTf, total transferrin and cholesterol were significantly lower in the AP group than that in the controls. In the chronic pancreatitis group, the mean values of GGT, ALP and CRP were significantly higher, but total transferrin was significantly lower in the CP group than that in the controls.

**Figure 1:**
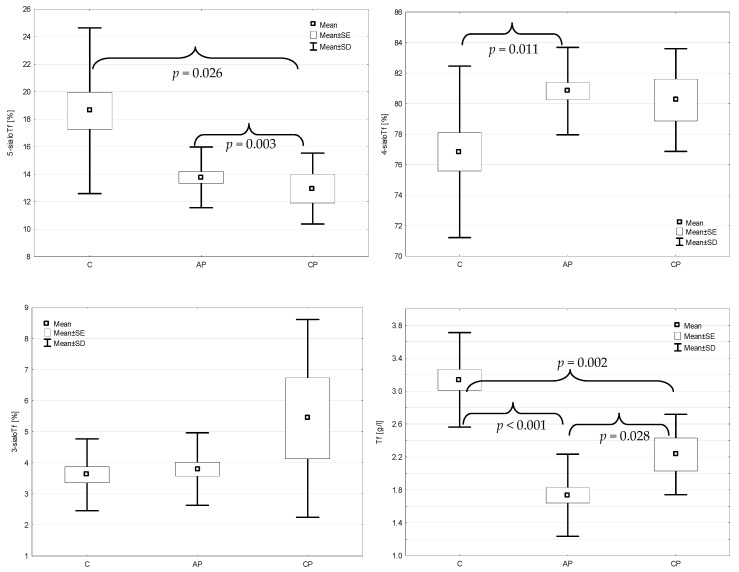
The concentrations of transferrin isoforms and total transferrin in the control (C), acute pancreatitis (AP) and chronic pancreatitis (CP) groups.

While testing the concentrations of transferrin isoforms, a statistically significant decrease in the 5-sialoTf concentrations in the acute and chronic pancreatitis groups was observed in comparison with the control group (*p* = 0.003 and *p* = 0.026, respectively) (Figure 1). Among the other isoforms of transferrin, only the concentration of 4-sialoTf was significantly higher in the acute pancreatitis group than that in the controls (*p* = 0.011). The concentration of 4-sialoTf, among other isoforms of transferrin, was exclusively one to be significantly higher in acute pancreatitis group than that in the controls. The concentrations of 3-sialoTf did not significantly change in both the (AP and CP) tested groups when compared to the control group (*p* = 0.722 and *p* = 273, respectively). There were also no significant differences in the concentrations of transferrin isoforms between the acute and chronic pancreatitis groups. Total transferrin concentrations were significantly lower in the AP and CP groups in comparison with the controls (*p* < 0.001 and *p* = 0.002, respectively) and in the acute pancreatitis group, it was significantly lower than that in the chronic pancreatitis group (*p* = 0.028).

**Figure 2:**
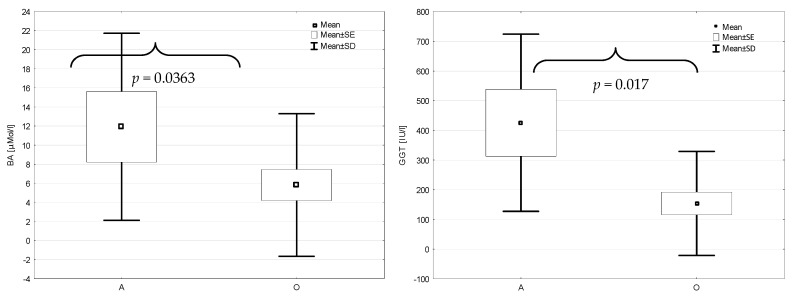
The concentration of bile acids (BA) and the activity of GGT in the acute pancreatitis of alcoholic (A) and other (O) etiologies.

Taking into consideration the morphological classification of acute pancreatitis, there were no statistically significant differences between edematous and necrotizing acute pancreatitis, also for carbohydrate-deficient transferrin (CDT) and other transferrin isoforms. Comparing the alcoholic and other etiologies of acute pancreatitis (Figure 2), there were significantly higher concentrations of bile acids (11.63 ± 9.46 µM/L) and the activity of GGT (338 ± 238 IU/L) in the alcoholic etiology of AP than that in the pancreatitis of other etiologies (5.75 ± 7.05 µM/L and 154 ± 175 IU/L, respectively) (*p* = 0.036 and *p* = 0.017, respectively).

## 4. Discussion

In this paper, we tried to determine the profile of transferrin isoforms in pancreatitis, in acute as well as in chronic forms of disease, in accordance with the knowledge on the changes of protein glycosylation in the course of inflammatory diseases. Because we previously assessed the profile of transferrin isoforms in many different diseases, including liver diseases, rheumatic diseases and pancreatic cancers, we have the opportunity to compare the changes in pancreatitis with the changes in the diseases mentioned above [6,10,11,13]. This situation created the possibility to show whether these changes are characteristic for pancreatitis or only organ-dependent inflammation. First, we found the changed profile of transferrin isoforms in pancreatitis, with more severe changes in acute pancreatitis. Thus, the concentration of pantasialotransferrin was decreased in both forms of pancreatitis, but the concentration of tetrasialotransferrin was only elevated in the acute form of pancreatitis. The concentration of trisialotransferrin was not changed and asialotransferrin was undetectable. As expected, the total transferrin concentration was diminished in both forms of pancreatitis, with an apparent greater decrease in acute pancreatitis. Looking at our earlier works, we can only see a similarity to the profile of transferrin isoforms in chronic hepatitis [13]. At the same time, we find differences in the transferrin isoform profile in other liver diseases [9]; in rheumatic diseases, including rheumatoid arthritis, systemic lupus erythematosus, systemic sclerosis and juvenile idiopathic arthritis [11,12,14]; and in pancreatic cancers [6]. Thus, we can conclude that the change in transferrin isoform profile in pancreatitis is only similar to that seen in hepatitis [13]. It is also different from that observed in pancreatic cancers [6]. In summary, the changes in serum transferrin isoform profile are not organ specific, but are characteristic for the pathogenesis of disease, in this case, inflammation.

As expected, we obtained a decreased total concentration of transferrin, with lower level in acute pancreatitis than that in the chronic one. It is a consequence of the nature of this glycoprotein as a negative protein of acute-phase reaction. From a diagnostic point of view, in many clinical situations, it is not sufficient to estimate only the total concentration of the measured compound, but also its components. This is especially true as far as enzymes (i.e., isoenzymes) and proteins (i.e., isoforms) are concerned. Therefore, the changes in the individual components of the whole system are of greater importance, regardless of the direction of shift of the entire system. Thus, individual isoforms that shift in varied directions can be observed. Moreover, it should be remembered that the results are expressed as a percentage. On the other hand, we do not have information about the absolute concentration of the isoforms. Therefore, it should not be interpreted that a reduction of about 30% of the isoform contribution in the total concentration is insufficient to reduce the total protein concentration by about 40%. These values cannot be compared with each other because they signify completely different things.

In this study, we expected changes in transferrin isoform profile due to the histological picture of pancreatitis, but we did not find differences between necrotic and edematous forms of inflammation. It may be because transferrin is mainly synthesized in the liver, and the effect of pancreatic inflammation on the liver is independent of the histological picture of pancreatic diseases [3]. However, we expected differences because, in the edematous form of pancreatitis, only the swelling and disruption of cytoplasmic organelles in the acinar cells occurs, but in necrotizing pancreatitis, the necrosis of the epithelial duct with periductal acute inflammation occurs [15]. The necrosis of cells should be accompanied by the release of the cells’ organelle content, such as proteins, glycoproteins, and enzymes. In this study, we did not observe any changes in all the studied laboratory tests, including transferrin isoforms, between the edematous and necrotizing forms of acute pancreatitis.

While testing the changes in other laboratory tests (Table 1), we did not see changes in bile acid concentration (due to the wide dispersion of results); however, taking into consideration the etiology of acute pancreatitis, we noticed significant differences. In acute pancreatitis of alcoholic etiology, the concentration of bile acids is higher than that in pancreatitis of other etiologies. We observed a similar change in the case of GGT, which seems to be an evident alteration considering the role of this enzyme as an indicator of alcohol abuse [16]. An increased level of bile acids in alcoholic liver diseases was confirmed in other studies [17,18].

In our opinion, it is necessary to distinguish the etiology of pancreatitis in the mixed group, especially in the group with obstructions in the biliary tract, as opposed to the other causes of inflammation (such as drugs and hypertriglyceridemia).

## 5. Conclusions

In conclusion, we showed the changes in transferrin isoform profile in acute and chronic pancreatitis, which are not organ specific, but specific to the pathogenesis of the disease, in this case, inflammation.

## Figures and Tables

**Table 1:** The characteristics of the controls and the acute and chronic pancreatitis groups.

Test	Controls (*n* = 30)	Acute Pancreatitis (*n* = 84)	Chronic Pancreatitis (*n* = 42)
MCV [fl]	87.1 ± 4.5	90.7 ± 5.8 *	88.0 ± 4.4
PLT [10^9^/L]	243.2 ± 55.1	258.1 ± 138.7	192.4 ± 66.7
PT [sec]	12.42 ± 0.42	13.04 ± 1.35	11.8 ± 1.5
INR	0.93 ± 0.04	1.09 ± 0.11 *	0.98 ± 0.12
CDT [%]	0.62 ± 0.19	1.53 ± 1.86 *	1.38 ± 2.07
5-sialoTf [%]	18.61 ± 6.03	13.76 ± 2.20 *	12.95 ± 2.58 *
4-sialoTf [%]	76.84 ± 5.62	80.82 ± 2.87 *	80.23 ± 3.36
3-sialoTf [%]	3.61 ± 1.16	3.80 ± 1.17	5.43 ± 3.18
Transferrin [g/L]	3.14 ± 0.57	1.74 ± 0.50 *#	2.23 ± 0.48 *
BA [µMol/L]	3.55 ± 2.14	10.75 ± 20.22	18.75 ± 28.34
Glucose [mM/L]	5.22 ± 0.42	5.56 ± 2.32	6.22 ± 2.33
AST [IU]	23.2 ± 5.3	70.6 ± 73.7 *	41.8 ± 20.8
ALT [IU/L]	17.6 ± 8.3	74.3 ± 79.1 *	57.5 ± 54.2
GGT [IU/L]	23.3 ± 7.3	221.7 ± 238.5 *	210.0 ± 168.2 *
Amylase [IU/L]	58.6 ± 14.3	627.7 ± 714.41 *#	94.5 ± 85.8
Lipase [IU/L]	36.4 ± 13.3	1263 ± 1931 *#	91.0 ± 118.1
ALP [IU/L]	61.4 ± 11.2	113.3 ± 110.3 *	194.5 ± 130.8 *
CRP [mg/L]	1.03 ± 0.78	96.0 ± 112.5 *#	4.5 ± 3.5 *
Bilirubin [µM/L]	12.48 ± 5.30	17.61 ± 11.6	11.45 ± 1.54
Cholesterol [mM/L]	5.13 ± 0.81	3.94 ± 1.18 *	4.77 ± 2.04
TG [mM/L]	1.12 ± 0.32	1.49 ± 0.92	1.43 ± 0.79

* Significant difference in comparison with the controls. # Significant difference in the comparison between AP and CP group.

## Data Availability

Data sharing not applicable.
